# Repeatability of global myocardial function parameters post pacemaker implantation

**DOI:** 10.1186/1532-429X-11-S1-P281

**Published:** 2009-01-28

**Authors:** Jana G Delfino, Jack A Talsma, Edward T Martin, David A Sandler, John N Oshinski

**Affiliations:** 1 Emory University, Atlanta, GA USA; 2 Oklahoma Heart Institute, Tulsa, OK USA; 3 Emory University/Georgia Institute of Technology, Atlanta, GA USA

**Keywords:** Pericarditis, Left Ventricular Volume, Steady State Free Precession, Constrictive Pericarditis, Reverse Remodel

## Objective

To determine the reproducibility of global myocardial function parameters in patients with implanted pacemakers.

## Introduction

Quantitative measurement of patient response to CRT includes reverse remodeling of the left ventricle. Usually this is measured by a >15% reduction in end-systolic left ventricular volume at 3 months post CRT. Two-dimensional echocardiography has been the primary modality used for this endpoint. There is an interest in using MRI to evaluate response because of its superior image quality, resolution and reproducibility compared to echo. However, the image quality and reproducibility of CMR in patient with dual chamber pacemakers is unknown.

## Methods

Six patients with implanted pacemakers and clinical indications for CMR were included in this study. All patients were not pacemaker dependent and had dual chamber devices (50% Medtronic Inc, 50% Guidant Inc). Clinical indications for CMR included pericardial mass, pre-pulmonary vein ablation, pulmonary vein stenosis, myocardial fibrosis and constrictive pericarditis.

MRI exams were performed on a 1.5 T GE Signa Excite MRI (GE Medical Systems**,** Waukesha, WI). Following acquisition of scout images, cine steady state free precession (SSFP) images were acquired in the two chamber, four chamber, and short axis orientations. Image acquisition parameters were as follows: TR = 3 msec, TE = 1.2 msec, slice thickness = 8 mm, FOV = 360–380 mm, pixel size 0.7–1.5 mm. Appropriate precautions were taken regarding pacemaker-MRI interaction including telemetry monitoring, voice and visual contact, and device interrogation pre/post MRI.

Endocardial and epicardial borders were traced on short axis cine SSFP images using MASS software (ARL, Lieden, The Netherlands). Global parameters of myocardial function [end diastolic volume (LVEDV), end systolic volume (LVESV), stroke volume (DV), ejection fraction (EF), and mass (LVM)] were computed from the contours. Complete border tracing and analysis was conducted by two independent observers on two separate days at least 1 week apart.

The mean difference between repeated measurements and the coefficient of variability (CV) were computed for each parameter to assess reproducibility.

## Results

**Figure 1:**
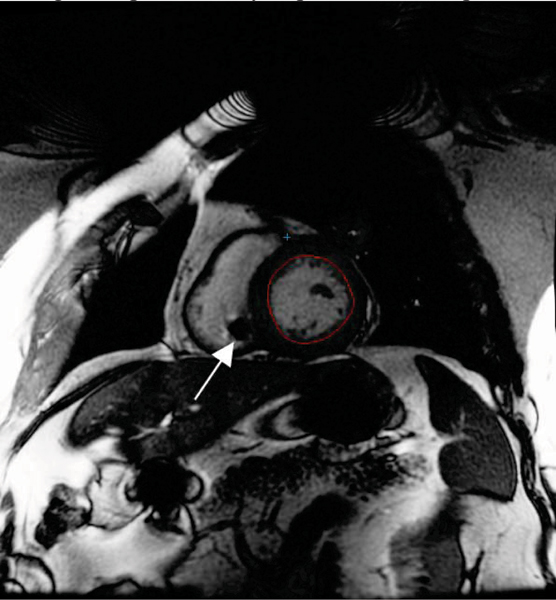
**Example CMR image from a patient with an implanted dual chamber pacemaker**. Although artifacts from both the pulse generator (top center) and the RV pacing lead are visible (arrow), image quality is sufficient for accurate analysis of myocardial function.

Image quality for all six patients was sufficient for analysis, Figure 1. Mean difference between repeated measurements was 15.6 +/- 6.9 mL for LVEDV, 6.9 +/- 7.6 mL for LVESV, 8.7 +/- 10.3 mL for SV, 0.7 +/- 6.3% for EF, and 3.1 +/- 9.2 g for LVEDM. Coefficient of variability was 7.9% for LVEDV, 7.8% for LVESV, 10.0% for SV, 6.2% for EF, and 4.9% for LVEDM.

## Discussion

The pulse generator and pacing leads caused signal void artifacts in the image. However, these were localized and did not interfere in identification of myocardial borders, Figure 1. Image quality decreased substantially in the one patient who had two additional abandoned leads from a previous device.

## Conclusion

Global measurements of myocardial function can be reliably measured with good reproducibility in patients with implanted dual chamber pacemakers.

